# Identifying individuals at risk of cognitive decline: cross-sectional analysis of variability in neuropsychological test scores among community-dwelling older adults

**DOI:** 10.1186/s12889-026-27246-y

**Published:** 2026-04-09

**Authors:** Lucía Sáez-González, Luis A. Martínez, Cristina García-García, G. Blázquez Abellán, José Antonio Carbajal de Lara, Rosa M. Martinez-Garcia, Lucía Castro-Vázquez

**Affiliations:** 1 NUTRISAF group, Faculty of Pharmacy, University of Castilla-La Mancha, Av. Dr. Jose María Sánchez Ibáñez s/n, Albacete, 02008 Spain; 2 Tiriez Community Pharmacy, Albacete, 02161 Spain; 3 Community Pharmacy Jose Antonio Carbajal de Lara, Albacete, Spain; 4 NUTRISAF group, Faculty of Nursing and Physiotherapy, University of Castilla-La Mancha, Cuenca, Spain; 5 Cátedra DeCo MICOF-CEU UCH, Universidad Cardenal Herrera-CEU, Valencia, Spain

**Keywords:** Cognitive impairment, Older adults, Community-dwellers, Neuropsychological tests, Screening, Prevalence, Hypercholesterolemia, Community pharmacy

## Abstract

**Background:**

Cognitive impairment is a major public health concern due to its impact on functional independence and its risk of progression to dementia. Early detection is critical, but the estimated prevalence varies substantially depending on the screening tool used and the role of modifiable metabolic risk factors, in accelerating cognitive aging.

**Objective:**

This study aimed to describe the variability in cognitive performance and the prevalence of low scores across several brief screening tools and cut-offs, and to explore sociodemographic, functional and metabolic factors associated with lower cognitive performance in community-dwelling older adults.

**Methods:**

A cross-sectional study was conducted with *N* = 286 community-dwelling participants aged over 60 years, recruited from community pharmacies in Albacete, Spain. Cognitive status was assessed using the MoCA (cut-offs < 26 and < 21), the Short Portable Mental Status Questionnaire, the Memory Impairment Screen, and the Semantic Verbal Fluency Test (animals). Comorbidities were assessed using active medication prescriptions as proxy variables. Cohen’s Kappa coefficients were computed to assess concordance, and a binary logistic regression was performed to identify potential predictors of cognitive impairment, defined as a MoCA score < 21.

**Results:**

The estimated prevalence of suspected cognitive impairment varied from 71.3% using the highest MoCA cut-off (< 26) to 25.2% using the more conservative MoCA < 21 threshold. Concordance analysis revealed low agreement between MoCA < 26 and the other instruments (Kappa < 0.08). However, using the MoCA < 21 cut-off, the observed agreement improved substantially to over 75% (all Kappa values statistically significant at *p* < 0.001). The adjusted binary logistic regression model demonstrated that older age significantly increased the odds of cognitive impairment (OR = 1.10, *p* < 0.001), whereas higher cognitive reserve was a protective factor (OR = 0.75, *p* < 0.001).

**Conclusions:**

The estimated prevalence of suspected cognitive impairment is highly dependent on the screening instrument and threshold selected. The findings support the adoption of a more conservative MoCA cut-off < 21 to enhance agreement with other brief instruments and may reduce potential overestimation of impairment. Additionally, the associations observed between metabolic conditions and lower cognitive performance highlight the importance of integrated preventive strategies in primary care, combining sensitive cognitive screening with cardiometabolic risk management.

**Supplementary Information:**

The online version contains supplementary material available at 10.1186/s12889-026-27246-y.

## Introduction

### Background

Cognitive impairment is a major public health concern due to its impact on functional independence, risk and progression to dementia, affecting quality of life and increasing healthcare needs and costs [1]. Early detection of cognitive decline is essential to implement preventive interventions and optimize care planning [2]. However, the selection of cognitive screening tools remains a matter of debate, as different instruments target partially overlapping cognitive domains and apply variable thresholds for impairment [3, 4].

After decades of using the Mini-Mental State Examination as the gold standard, the Montreal Cognitive Assessment (MoCA) has been widely adopted as a screening tool with high sensitivity for mild cognitive impairment, encompassing a broad range of domains including memory, executive function (not assessed in Mini-Mental State Examination), attention, language, and visuospatial abilities [3, 5]. In addition to the widespread use of the MoCA as a broad multi-domain screener, several brief instruments are routinely applied in community and primary-care-oriented programmes to classify older adults as screen-negative or screen-positive for suspected cognitive impairment. The MoCA is a multidomain screening tool covering memory, executive functions, attention, language and visuospatial abilities, and it is known for its high sensitivity to mild cognitive impairment [6]. In contrast, other brief instruments such as the Short Portable Mental Status Questionnaire (SPMSQ) that focuses mainly on orientation, short-term memory and basic calculation; the Memory Impairment Screen (MIS) which assesses episodic memory through cued recall; and semantic verbal fluency tasks (SVF) that primarily tap lexical access and executive retrieval processes; are more targeted in scope [2]. Although these instruments do not assess identical cognitive domains, they are frequently used in primary care and community settings as alternative or complementary tools. Examining the extent to which a broad global screener such as the MoCA converges with brief, domain-focused tools is therefore relevant to understand how methodological choices influence estimates of suspected impairment in non-clinical populations. Furthermore, the cut-off point used to define impairment also substantially influences prevalence estimates, with studies in Spanish populations recommending lower thresholds (e.g. MoCA < 21, <23) to improve specificity and reduce false positives [4, 7–10].

In this study, the SPMSQ, MIS and SVF (animals) were selected because they are the tools used in the *Cátedra DECO (CEU–MICOF)* community pharmacy initiative, and were chosen by neurologists from the Valencian Health Service as pragmatic screening instruments in non-clinical settings [11]. Despite the previously stated fact that these instruments do not evaluate analogous cognitive domains, they are unified by a shared objective in public health, namely the identification of individuals who may require further clinical evaluation. Consequently, a comparison of their screening classifications with those derived from the MoCA is informative, as it enables an understanding of the influence of instrument selection and cut-offs on the estimated prevalence and case classification in community-dwelling populations.

Importantly, the screening consistency among these tools has been insufficiently characterized in community-dwelling older adults. Understanding the degree of agreement and the potential determinants of test performance is critical to guide screening strategies in primary care and research settings.

In addition to the selection of cognitive screening tools, growing evidence highlights the relevance of addressing modifiable risk factors throughout the life course. The Lancet Commission on Dementia Prevention, Intervention, and Care has identified 14 potentially modifiable risk factors that could prevent or delay nearly half of dementia cases worldwide [12]. These span from ensuring access to quality education and cognitively stimulating activities in early and midlife, to managing hearing and vision loss, and effectively treat depression. Lifestyle-related interventions, such as encouraging regular physical activity, reducing toxic habits as smoking and alcohol consumption and promoting social participation, are also essential in reducing vulnerability to cognitive decline. Crucially, a cluster of metabolic and cardiovascular risk factors—including hypertension, dyslipidemia, diabetes and obesity—has been consistently associated with accelerated cognitive aging and higher dementia risk [13–16]. Among these, detecting and treating elevated LDL-cholesterol from midlife, maintaining systolic blood pressure ≤ 130 mmHg, and preventing obesity play a pivotal role in preserving cognitive health. Addressing these metabolic conditions is particularly relevant in community-dwelling older adults, where multimorbidity and polypharmacy are prevalent, and early detection may offer opportunities for integrated interventions combining cardiovascular risk management with cognitive screening strategies [12–14].

### Study objectives

The primary objective of this study was to describe how different brief cognitive screening tools (MoCA, MIS, SPMSQ, SVF) classify community-dwelling older adults when alternative cut-offs are applied (MoCA < 26 vs. MoCA < 21), and to examine the variability in cognitive performance across instruments in a non-clinical population without a reference diagnostic standard of cognitive impairment. A secondary objective was to explore factors associated with lower cognitive scores. Factors explored included sociodemographic variables, cognitive reserve, functional independence and comorbidities, amongst others [10, 17, 18]. Special attention was paid to the role of metabolic conditions, such as hypercholesterolemia, diabetes and hypertension, which have been implicated in cognitive aging [13, 14, 16].

## Materials and methods

### Study design and participants

A cross-sectional study was conducted including community-dwelling participants older than 60 years of age, recruited from community pharmacies in the province of Albacete, Spain from April 2024-December 2025.

Inclusion criteria were: (a) age older than 60; (b) non-institutionalized; (c) absence of significant functional or sensorial impairment, or any major difficulty to complete the interview; (d) Spanish-native speaker.

Exclusion criteria were: (a) presence of sensorial or functional impairment or any major difficulty to complete the interview; (b) history of stroke, neurological, or conditions that could interfere on cognitive performance.

Eligibility criteria were assessed by the community pharmacists participating in the study. Potential participants were initially screened for inclusion and exclusion criteria at the pharmacy, and those who did not meet the criteria were excluded before scheduling the interview. Only individuals who fulfilled all criteria and agreed to participate were invited to attend the full assessment session.

The minimum required sample size was estimated based on an expected prevalence of cognitive impairment of 11.6%, as reported in the DERIVA study [19]. Using a 95% confidence level, the calculated minimum sample size was 158 participants. A total of 365 participants were initially recruited for the study; however, only 286 met all inclusion criteria and completed the full assessment protocol.

### Human ethics and consent to participate

The research protocol was approved by the Ethics Committee of the Albacete Integrated Care Management System (approval reference: 2023 − 155) and was conducted in accordance with the Declaration of Helsinki. All participants voluntarily enrolled the study and signed written informed consent.

### Measures

Participants were invited to attend an individual face-to-face interview with a trained pharmacist for an approximate duration of 90 min, with the purpose of collecting all the relevant information.

#### Sociodemographic variables

The following variables were obtained: age, sex and rural or urban residence by postal code.

#### Cognitive assessment

Cognitive status was evaluated using four neuropsychological tests validated for Spanish-populations:

- *Spanish version of the Montreal Cognitive Assessment (MoCA)*
  Global cognitive screening tool assessing all cognitive domains (0–30). Two cut-off points were used to define suspected cognitive impairment: <26, as proposed in the original publication, and < 21, following recommendations by Lozano-Gallego et al. [20] and other studies to improve specificity in older populations [9, 10].
- *Short Portable Mental Status Questionnaire (SPMSQ) of Pfeiffer*
  A brief screening instrument with 10 items assessing orientation, memory and calculation. Scores ≥ 3 errors were considered suggestive of possible cognitive impairment [21, 22].
- *Memory Impairment Screen (MIS)*
  Test of episodic memory consisting in a 4-item delayed free and cued recall (0–8). Scores ≤ 4 were considered suggestive memory impairment [23, 24].
- *Semantic Verbal Fluency Test (SVF) – Animals*
  Participants were asked to name as many animals as possible in 60 s. A score below 10 was considered indicative of impairment in Spanish older adults [25] .

The MoCA was used as a broad multi-domain cognitive screener. The SPMSQ, MIS and SVF were included because they are brief tools commonly implemented in community/primary care-oriented screening initiatives and are part of the standard cognitive screening battery used in the Cátedra DECO (CEU–MICOF) programme, selected by neurologists from the Valencian Health Service. These instruments target partially overlapping constructs (e.g., orientation/attention, episodic memory, and semantic fluency/executive retrieval) and were compared at the level of screening classifications and total scores rather than as interchangeable domain-equivalent measures.

Cognitive tests were applied in a fixed order: (i) MIS-learning of the words; (ii) SPMQS-used as distractor from the MIS; (iii) MIS-free or cued recall of the words; (iv) SVF-animals; (v) MoCA.

#### Cognitive reserve

Cognitive reserve was assessed using the validated Spanish-version of the *Cuestionario de Reserva Cognitiva* (CRC) (Rami et al.., 2011), which includes items on formal educational level, occupational complexity, and engagement in cognitive leisure activities: music, reading, speaking different languages and doing mental puzzles or games. The total score ranges from 0 to 25, with higher scores indicating greater cognitive reserve [26–28].

#### Medication review and comorbidity assessment

Medication data were collected through structured interview and review of medical prescriptions. For each participant, the following were recorded: (i) active principles (ATC code); (ii) daily dose; (iii) number of concurrent medications; (iv) polypharmacy status (defined as ≥ 5 active principles). Comorbidities were inferred from current pharmacological treatment, using medications as proxy variables for comorbidities; thus, a diagnosis was assumed if the participant was taking medications intended specifically to treat that disease. Specifically, hypertension, diabetes or dyslipidemia were assumed if the participant was taking any kind of anti-hypertensives, glucose-lowering agents or lipid-lowering drugs, respectively. In addition, comorbidities were cross-checked through self-reported medical history during the medication review section of the interview, and, in case of doubt, medical records provided by the participant were consulted. Nevertheless, we acknowledge that this proxy approach may not capture undiagnosed or untreated cases and may misclassify conditions when medications are prescribed for alternative indications.

#### Functional independence

Independence in basic activities of daily living (ADL) was assessed with the Barthel Index (range 0-100), where higher scores indicate greater independence; scores ≥ 90 are generally considered compatible with functional independence in basic ADL. Instrumental ADL were evaluated using the Lawton and Brody Index (range 0–8), with higher scores reflecting greater independence; scores of 7–8 typically indicate preserved instrumental ADL function.

#### Interview protocol

Participants attended an individual face-to-face interview in a quiet area of the community pharmacy, lasting approximately 90 min. All assessments were conducted by trained community pharmacists following a standardized protocol.

A total of five community pharmacists took part in data collection. Before recruitment started, they received standardized training on test administration and scoring, which included written instructions and several supervised pilot interviews to ensure consistency across assessors.

After a brief introduction to the study aims and procedures, the interview commenced following the next fixed structure: first sociodemographic data were collected, followed by cognitive tests in a fixed order: (i) MIS-learning of the words; (ii) SPMQS-used as distractor from the MIS; (iii) MIS-free or cued recall of the words; (iv) SVF-animals; (v) MoCA; next medication review and independence assessment were conducted, and last a cognitive reserve questionnaire was administered. Short breaks were allowed as needed to minimize fatigue, but never in the middle of a cognitive test or between the MIS and the SPMSQ.

Participants were informed that the assessment did not constitute a formal diagnosis, but they could receive a brief summary of their performance if requested. In cases of clearly abnormal results suggesting possible cognitive impairment, pharmacists encouraged participants to consult their primary care physician and provided a written note summarizing the findings.

### Statistical analysis

In addition to descriptive statistics, concordance analyses and further *post-hoc* comparisons were performed to explore associations between cognitive impairment and participant characteristics.

Two binary variables were created for MoCA classification (< 26 and < 21), and cross-tabulations were performed to compare prevalence and agreement across the instruments. Cohen’s Kappa coefficients were computed to assess concordance between MoCA and the other tests under each cut-off criterion.

A bivariate correlation analysis was also conducted to evaluate the relationships among the total scores obtained on the different cognitive screening tests using Pearson’s correlation coefficient. Statistical significance was set at *p* < 0.05. Two-tailed tests were applied.

Categorical variables (sex, rural, chronic conditions, polypharmacy) were compared using chi-square tests and Odds Ratios (OR) with 95% confidence intervals (CI) were calculated when applicable.

Pearson correlation coefficients were calculated to explore the relationship between continuous variables such as age, cognitive reserve (CRC), number of medications, independence in basic and instrumental activities of daily living (Barthel Index and Lawton and Brody Scale) and cognitive test scores.

To identify possible predictors of cognitive impairment, binary logistic regression analysis was conducted including sociodemographic, clinical, and functional variables as potential covariates. The dependent variable was cognitive impairment, defined as a MoCA score < 21. Results were reported as OR with 95% CI.

Linear regression analyses were also performed using the different neuropsychological test’s total score as the dependent variables, to further explore associations with potential predictors and cognitive performance between tests. Separate regression models were estimated for each cognitive test.

All statistical analyses were performed using IBM SPSS Statistics v29 (IBM Corp.), with statistical significance set at *p* < 0.05 (two-tailed).

Given the sample size and the number of potential predictors, multivariable models were considered exploratory. To reduce the risk of overfitting, only variables with a p-value < 0.05 in univariate analyses were considered for inclusion in the multivariable models. The resulting odds ratios and regression coefficients should therefore be interpreted with caution as hypothesis-generating estimates.

Missing data were assessed prior to analysis. The proportion of missing data was minimal; however, participants with missing values in any of the variables required for the main analyses were excluded from the final analytical sample. All analyses were conducted on complete cases without imputation.

## Results

### Sample characteristics

A total of 286 participants participated in the study with a mean age of 73.25 ± 6.479, and 64% were women (183). The most frequent comorbidity was hypercholesterolemia (58.0%), followed by hypertension (52.1%). More than half of the participants (55.6%) met criteria for polypharmacy (≥ 5 medications). Cognitive reserve averaged 10.97 ± 5.065 points, and most participants were functionally independent (mean Barthel Index 98.31 ± 5.698; Lawton and Brody 7.65 ± 1.011). Full descriptive characteristics of the sample overall and by sex are shown in Table 1.

**Table 1:** Main characteristics of the sample

Characteristics	Total	Men	Women
Number of subjects N	286 (100%)	103 (36.0%)	183 (64.0%)
Age Mean (SD) (min-max)	73.25 (6.479) (60.50-92.32)	73.34 (6.973) (60.97–89.95)	73.19 (6.638) (60.50-92.32)
60–69	108 (37.8%)	39 (37.9%)	69 (37.7%)
70–79	120 (42.0%)	42 (40.8%)	78 (42.6%)
≥ 80	58 (20.3%)	22 (21.4%)	36 (19.7%)
Rural N (%)	65 (22.7%)	20 (19.4%)	45 (24.6%)
Comorbidities N (%)			
Hypertension	149 (52.1%)	57 (55.3%)	92 (50.3%)
Hypercholesterolemia	166 (58.0%)	57 (55.3%)	109 (59.6%)
Type 2 Diabetes Mellitus	61 (21.3%)*	29 (28.2%)	32 (17.5%)
Medication N (%)			
Antidepressants (N06A)	38 (13.3%)*	7 (6.8%)	31 (16.9%)
Anxiolytics, Hypnotics (N05B, N05C)	60 (21.0%)*	14 (13.6%)	46 (25.1%)
Dementia (Donepezile)	2 (0.7%)	1 (0.3%)	1 (0.3%)
Cognitive Test Mean (SD) (min-max)			
SPMSQ (0–11)	1.01 (1.185)**(0–8)	0.77 (1.131)(0–6)	1.15 (1.195)(0–8)
MIS (0–8)	7.21 (1.209)(2–8)	7.18 (1.183)(4–8)	7.22 (1.227)(2–8)
SVF (Animals) (0-∞)	18.14 (5.776)***(5–37)	19.74 (5.697)(6–32)	17.25 (5.639)(5–37)
MoCA (0–30)	22.75 (4.775)(6–31)	23.06 (4.331)(6–30)	22.58 (5.010)(6–31)
Cognitive Reserve Mean (SD) (min-max)	10.97 (5.065) (0–23)	11.62 (4.847) (0–21)	10.60 (5.160) (0–23)
Number of active principles Mean (SD) (min-max)	5.94 (4.126)(0–22)	5.62 (3.934)(0–15)	6.12 (4.230)(0–22)
Polypharmacy N (%)	159 (55.6%)	53 (51.5%)	106 (57.9%)
Independence Mean (SD) (min-max) Barthel Index	98.31 (5.698)* (55–100)	99.27 (4.339) (70–100)	97.76 (6.282) (55–100)
Lawton and Brody	7.65 (1.011)**(1–8)	7.40 (1.324)(1–8)	7.79 (0.751)(1–8)

*MIS* Memory Impairment Screen, *MoCA* Montreal Cognitive Assessment, *SPMSQ* Short Portable Mental Status Questionnaire (numbers of errors), *SVF* Semantic Verbal Fluency. Significance level at: *: *p* < 0.05; **: *p* < 0.01; ***: *p* < 0.001

### Cognitive impairment assessment: prevalence, concordance and correlation

Regarding cognitive assessment, the average score in the Montreal Cognitive Assessment (MoCA) was 22.75 ± 4.775 (Table 1). When applying a cut-off of < 26, 204 participants (71.3%) screened positive for cognitive impairment. This percentage dropped to 25.2% using the more conservative cut-off of < 21 (Table 2).

**Table 2:** Prevalence of CI with different tests and cut-off points by sex and age groups

Test	Cut-off	*N* (%) positive											
		Total				Men				Women			
		60–69	70–79	≤ 80	Total	60–69	70–79	≤ 80	Total	60–69	70–79	≤ 80	Total
MoCA	< 26	64 (59.3%)	89 (74.2%)	51 (87.9%)	204 (71.3%)***	26 (66.7%)	32 (76.2%)	21 (95.5%)	79 (76.7%)	38 (55.1%)	57 (73.1%)	30 (83.3%)	125 (68.3%)
	< 21	12 (11.1%)	30 (25.0%)	30 (51.7%)	72 (25.2%)***	2 (5.1%)	10 (23.8%)	11 (50.0%)	23 (22.3%)	10 (14.5%)	20 (25.6%)	19 (52.8%)	49 (26.8%)
MIS	≤ 4	3 (2.8%)	8 (6.7%)	6 (10.3%)	17 (5.9%)	0 (0.0%)	4 (9.5%)	3 (13.6%)	7 (6.8%)	3 (4.3%)	4 (5.1%)	3 (8.3%)	10 (5.5%)
SPMSQ	≥ 3	2 (1.9%)	9 (7.5%)	16 (27.6%)	27 (9.4%)***	0 (0.0%)	5 (11.9%)	4 (18.2%)	9 (8.7%)	2 (2.9%)	4 (5.1%)	12 (33.3%)	18 (9.8%)
SVF	< 10	1 (0.9%)	3 (2.5%)	9 (15.5%)	13 (4.5%)***	0 (0.0%)	1 (2.4%)	1 (4.5%)	2 (1.9%)	1 (1.4%)	2 (2.6%)	8 (22.2%)	11 (6.0%)

*MIS* Memory Impairment Screen, *MoCA* Montreal Cognitive Assessment, *SPMSQ* Short Portable Mental Status Questionnaire, *SVF* Semantic Verbal Fluency. ***: Significance at *p* < 0.001 level

The prevalence of cognitive impairment varied substantially across screening tools: 9.4% for Short Portable Mental Status Questionnaire (SPMSQ), 5.9% for Memory Impairment Screen (MIS), and 4.5% for Semantic Verbal Fluency (SVF), compared to a 71.3% with MoCA (cut-off score of < 26) (Table 2). When stratified by age groups, the prevalence of suspected cognitive impairment increased with age in both men and women in all tests. Slightly higher proportions of women classified as impaired were observed, although differences were not statistically significant. 

Concordance analysis (Table 3) revealed low agreement between MoCA cut-off score < 26 and the other tests (observed agreement between 32.5% and 38.11%, all Kappa < 0.08, *p* < 0.05). However, when using MoCA < 21 as a cut-off, the concordance improved, with observed agreement > 75% and statistically significant Kappa values (*p* < 0.001 in all cases).

**Table 3:** Concordance analysis between MoCA and alternative cognitive screening tests (MIS, SPMSQ, SVF)

Comparison	Positive cases (%)	Negative cases (%)	Observed agreement (%)	Kappa (95%)	*p*-value
MoCA < 26 vs. MIS vs. SPMSQ vs. SVF	125 (68.3%) 10 (5.5%) 18 (9.8%) 11 (6.0%)	161 (31.7%) 276 (94.5%) 268 (90.2%) 275 (94.0%)	33.92% 38.11% 32.52%	0.0394 0.0804 0.0275	0.016* < 0.001*** 0.043*
MoCA < 21 vs. MIS vs. SPMSQ vs. SVF	49 (26.8%) 10 (5.5%) 18 (9.8%) 11 (6.0%)	237 (73.2%) 276 (94.5%) 268 (90.2%) 275 (94.0%)	75.87% 81.47% 78.67%	0.1422 0.3794 0.2225	< 0.001*** < 0.001*** < 0.001***

*MIS* Memory Impairment Screen, *MoCA* Montreal Cognitive Assessment, *SPMSQ* Short Portable Mental Status Questionnaire, *SVF* Semantic Verbal Fluency

The correlation analysis demonstrated statistically significant associations among all cognitive tests evaluated (Appendix 1). The total MoCA score showed a moderate negative correlation with the SPMSQ (*r* = −0.6048; *p* < 0.001), indicating that higher MoCA scores were associated with fewer errors on the SPMSQ. Furthermore, MoCA scores were positively correlated with the MIS (*r* = 0.3510; *p* < 0.001) and the SVF (*r* = 0.4741; *p* < 0.001), suggesting that better global cognitive performance was related to greater recall and verbal fluency. The SPMSQ was negatively correlated with the MIS (*r* = −0.3645; *p* < 0.001) and the SVF (*r* = −3849; *p* < 0.001). Finally, a positive correlation was also observed between MIS and SVF scores (*r* = 0.2474; *p* = 0.001).

Given the different domain emphasis and intended scope of each tool, agreement metrics were interpreted as concordance between screening classifications rather than as evidence of diagnostic equivalence.

### Participant characteristics affecting test results

Associations between sociodemographic and clinical variables of participant characteristics and cognitive impairment, as assessed by each test, are presented in Table 4. For categorical variables, chi-square tests were performed using dichotomized cognitive impairment outcomes, and the percentage of positive suspected cognitive impairment is represented for each group. For continuous variables, Pearson’s correlation coefficients were calculated with the total test scores.

**Table 4:** Associations between participant characteristics and cognitive impairment according to different screening tools

Variables *N* = 286	MoCA		MIS PC = 17	SVF PC = 13	SPMSQ PC = 27
	Cut-off < 26 PC = 204	Cut-off < 21 PC = 72			
Sex/Gender n/n (%^a^)					
Male	79/103 (76.70%)	23/103 (22.33%)	7/103 (6.80%)	2/103 (1.94%)	9/103 (8.74%)
Female	125/183 (68.31%)	49/183 (26.78%)	10/183 (5.46%)	11/183 (6.01%)	18/183 (9.84%)
Chi^2^	2.2701	0.6915	0.2090	2.5151	0.0930
Age					
Pearson’s r	−0.4207***		−0.2121***	−0.3546***	0.3254***
Rural n/n (%^a^)					
No	152/221 (68.78%)	46/221 (20.81%)	13/221 (5.88%)	8/221 (3.62%)	17/221 (7.69%)
Yes	52/65 (80.00%)	26/65 (40.00%)	4/65 (6.15%)	5/65 (7.69%)	10/65 (15.38%)
Chi^2^	0.305	9.8146	0.0066	1.9198	3.4763
Hypertension n/n (%^a^)					
No	90/137 (65.69%)	27/137 (19.71%)	5/137 (3.65%)	2/137 (1.46%)	7/137 (5.11%)
Yes	114 (76.51%)	45 (30.20%)	12 (8.05%)	11 (7.38%)	20 (13.42%)
Chi^2^	4.0833*	4.1721*	2.4762	5.7704*	5.7698*
Hypercholesterolemia n/n (%^a^)					
No	76/120 (63.33%)	23/120 (19.17%)	7/120 (5.83%)	2/120 (1.67%)	7/120 (5.83%)
Yes	128/166 (77.11%)	49/166 (29.52%)	10/166 (6.02%)	11/166 (6.63%)	20/166 (12.05%)
Chi^2^	6.4625*	3.9619*	0.0045	3.9490 *	3.1467
Diabetes n/n (%^a^)					
No	157/225 (69.78%)	50/225 (22.22%)	10/225 (4.44%)	9/225 (4.00%)	18/225 (8.00%)
Yes	47/61 (77.05%)	22/61 (36.07%)	7/61 (11.48%)	4/61 (6.56%)	9/61 (14.75%)
Chi^2^	1.2407	4.8822*	4.2433*	0.395	2.5606
Depression n/n (%^a^)					
No	171/248 (68.95%)	58/248 (23.39%)	14/248 (5.65%)	11/248 (4.44%)	24/248 (9.48%)
Yes	33/38 (86.84%)	14/38 (36.84%)	3/38 (7.89%)	2/38 (5.26%)	3/38 (7.89%)
Chi^2^	5.1571*	3.1668	0.2983	0.0520	0.1225
Polypharmacy n/n (%^a^)					
No	81/127 (63.78%)	22/127 (17.32%)	3/127 (2.36%)	2/127 (1.57%)	8/127 (6.30%)
Yes	123/159 (77.36%)	50/159 (31.45%)	14/159 (8.81%)	11/159 (6.92%)	19/159 (11.95%)
Chi^2^	6.3658*	7.4768***	5.2423*	4.6462*	2.6368
Number of AP					
Pearson’s r	−0.2418***		−0.2098***	−0.1644***	0.1330*
Cognitive reserve					
Pearson’s r	0.5693***		0.2313*	0.4617***	−0.4163***
Independence					
Barthel Index Pearson’s r	0.3301***		0.2241***	0.2313***	−0.3942***
Lawton & Brody Pearson’s r	0.2383***		0.2320***	0.1998***	−0.2888***

*MIS* Memory Impairment Screen, *MoCA* Montreal Cognitive Assessment, *PC* Positive cases, *SPMSQ* Short Portable Mental Status Questionnaire, *SVF* Semantic Verbal Fluency

*: *p* < 0.05; **: *p* < 0.01; ***: *p* < 0.001

^a^row-based percentages

Age emerged as a consistent correlate of poorer cognitive performance across all tests. Older participants showed lower MoCA, MIS and SVF scores and more errors on the SPMSQ, with moderate correlation coefficients and statistically significant associations -MoCA (*r* = −0.4207, *p* < 0.001); MIS (*r* = −0.2121, *p* < 0.001); SVF (*r* = −0.3546, *p* < 0.001); SPMSQ errors (*r* = 0.3254, *p* < 0.001). Furthermore, the comparison of test results by age groups (Table 2) revealed that participants in the oldest age group had markedly lower MoCA and SVF scores and more errors in the SPMSQ than those aged 60–69 years, highlighting the pervasive influence on age on multiple cognitive domains in this community sample.

Female sex had a higher proportion of participants classified as cognitively impaired by the MoCA < 26 cut-off (68.31%), the MoCA < 21 cut-off (26.78%), the SVF (6.01%) and the MIS (5.46%), although chi-square tests did not reach statistical significance for these comparisons.

Regarding comorbidities, the presence of metabolic conditions was found to be associated with probable cognitive impairment detected by all tests. In particular, hypercholesterolemia was significantly associated with MoCA impairment with both cut-offs (cut-off < 26: chi^2^ = 6.4625, *p* = 0.01 and cut-off < 21: chi^2^ = 3.9619, *p* = 0.047), and also with impairment identified by the SVF (chi^2^ = 3.9490, *p* = 0.047). Hypertension was associated with possible cognitive impairment detected by both MOCA cut-offs (cut-off < 26: chi^2^ = 4.0833, *p* = 0.043 and cut-off < 21: chi^2^ = 4.1721, *p* = 0.041), SVF (chi^2^ = 5.7704, *p* = 0.016) and SPMSQ (chi^2^ = 5.7698, *p* = 0.016), but no significant associations were observed for the MIS. Regarding diabetes, associations were only observed with possible impairment detected by MoCA < 21 (chi^2^ = 4.8822, *p* = 0.027) cut-off and MIS (chi^2^ = 4.2433, *p* = 0.039).

Cognitive reserve showed positive correlations with MoCA (*r* = 0.5693, *p* < 0.001), MIS (*r* = 0.2313, *p* < 0.001), and SVF (*r* = 4617, *p* < 0.001), and a negative correlation with SPMSQ errors (*r* = −0.4163, *p* < 0.001).

Concerning functional measures, higher independence in basic activities of daily living, measured by the Barthel Index, was associated with better performance in all tests. With regard to the Lawton and Brody scale, a higher degree of independence in instrumental activities of daily living was found to be significantly associated with better cognitive tests scores measured by all tests, with the exception of MoCA < 26 cut-off.

Polypharmacy was associated with suspected cognitive impairment detected by all tests except for SPMSQ (MoCA < 26: chi^2^ = 6.3658, *p* = 0.012; MoCA < 21: chi^2^ = 7.4768, *p* = 0.006; MIS: chi^2^ = 5.2423, *p* = 0.022; and SVF: chi^2^ = 4.6462, *p* = 0.031). The number of chronic medications also showed association with performance in all cognitive tests (MoCA < 26: *r* = −0.2418, *p* < 0.0001; MIS: *r* = −0.2098, *p* < 0.0001; SVF: *r* = −0.1644, *p* = 0.0053; and SPMSQ: *r* = 0.1330, *p* = 0.0245).

### Regression models

A binary logistic regression analysis was conducted to examine the association between possible cognitive impairment detected by MoCA < 21 and potential predictors, including age, sex, cognitive reserve, rural residence, independence, polypharmacy and metabolic conditions (*p* < 0.05 in univariate analysis). The logistic regression model was statistically significant, which supports the influence of the predictors on the likelihood of exhibiting cognitive impairment. The model’s constant was B = −7.489 (SE = 0.002, *p* = 0.05).

Age was positively associated with lower cognitive scores (OR = 1.10, SE = 0.030, *p* < 0.001). This indicates that each additional year of age increased the odds of cognitive impairment by 10%.

Cognitive reserve was inversely associated with possible cognitive impairment detected by MoCA < 21 (OR = 0.75, SE = 0.038, *p* < 0.001), suggesting that higher cognitive reserve reduced the odds by approximately 25% per unit increase.

Importantly, associations with metabolic conditions, polypharmacy, independence and rural residence, observed in univariable analyses, were attenuated and no longer statistically significant after adjustment by age and cognitive reserve, suggesting confounding by these factors.

**Fig. 1:**
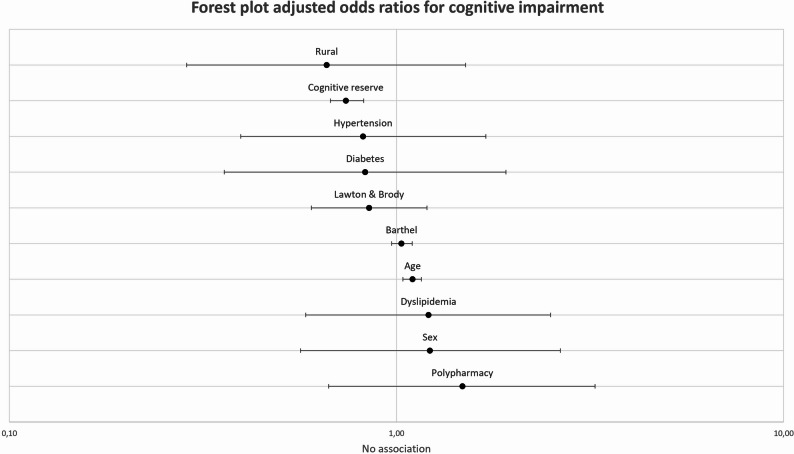
Forest plot adjusted odds ratios for cognitive impairment. Bars represent 95% confidence intervals. Cognitive impairment is defined by MoCA < 21

A forest plot was generated to illustrate the effect sizes of each predictor, showing odds ratios and 95% confidence intervals (Fig. 1).

**Fig. 2:**
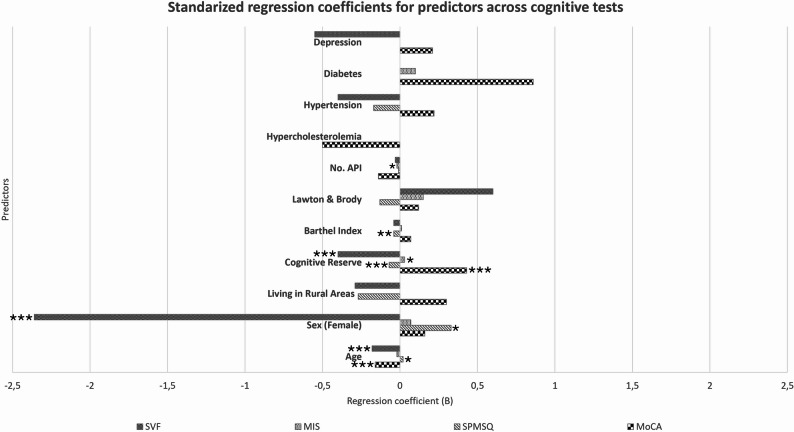
Standardized regression coefficients for predictors across cognitive tests. MIS: Memory Impairment Screen; MoCA: Montreal Cognitive Assessment; SPMSQ: Short Portable Mental Status Questionnaire; SVF: Semantic Verbal Fluency. Positive values indicate an association with higher test scores (better cognitive performance), while negative values indicate an association with lower scores. For the SPMSQ, higher scores reflect more errors and worse performance. Bars represent the estimated B coefficients. Non-significant predictors are included for comparison. Coefficients significance *: *p*<0.05; **: *p*<0.01; ***: *p*<0.001

Separate linear regression models were estimated to examine the predictors of performance in each cognitive test (Table 5). Variables with *p* < 0.05 in univariate models were added to the multivariable models. Standardized regression coefficients for predictors across cognitive tests are shown in Fig. 2.

**Table 5:** Linear regression models for each cognitive test performance

Test	Predictor	B	*p*-value	Adj *R*²
MoCA	Age	−0.161	< 0.001	0.386
	Sex (Female)	+ 0.164	0.739	
	Living in Rural Areas	+ 0.300	0.617	
	Cognitive Reserve	+ 0.428	< 0.001	
	Barthel Index	+ 0.074	0.133	
	Lawton and Brody	+ 0.115	0.673	
	No. of Active Drugs	−0.135	0.052	
	Hypercholesterolemia	−0.495	0.310	
	Hypertension	+ 0.217	0.661	
	Diabetes	+ 0.860	0.177	
	Depression	+ 0.211	0.764	
SPMSQ	Age	+ 0.024	0.016	0.277
	Sex (Female)	+ 0.333	0.012	
	Living in Rural Areas	−0.268	0.096	
	Cognitive Reserve	−0.071	< 0.001	
	Barthel Index	−0.040	0.002	
	Lawton and Brody	−0.129	0.074	
	No. of Active Drugs	−0.009	0.572	
	Hypertension	−0.170	0.203	
MIS	Age	−0.015	0.170	0.099
	Sex (female)	+ 0.073	0.629	
	Cognitive Reserve	+ 0.032	0.033	
	Barthel Index	+ 0.014	0.360	
	Lawton and Brody	+ 0.154	0.066	
	No. of Active Drugs	−0.015	0.019	
	Diabetes	+ 0.099	0.606	
SVF	Age	−0.177	< 0.001	0.278
	Sex (Female)	−2.361	< 0.001	
	Living in Rural Areas	−0.289	0.712	
	Cognitive Reserve	−0.404	< 0.001	
	Barthel Index	−0.039	0.544	
	Lawton and Brody	+ 0.597	0.089	
	No. of Active Drugs	−0.031	0.698	
	Hypertension	−0.395	0.544	
	Depression	−0.552	0.549	

*Adj R*^*2*^ Adjusted R^2^, *B* Regression constant, *MIS* Memory Impairment Screen, *MoCA* Montreal Cognitive Assessment, *SPMSQ* Short Portable Mental Status Questionnaire, *SVF* Semantic Verbal Fluency

#### Montreal Cognitive Assessment (MoCA)

For the MoCA total score, older age was significantly associated with lower scores (B = −0.161, *p* < 0.001), while higher cognitive reserve was associated with higher scores (B = + 0.428, *p* < 0.001). Living in rural areas, metabolic conditions, depression and independence showed nonsignificant associations. The model explained 38.6% of the variance (adjusted R²=0.386).

#### Short Portable Mental Status Questionnaire (SPMSQ)

In the SPMSQ model, older age (B = + 0.024, *p* = 0.016), and lower cognitive reserve (B = −0.071, *p* < 0.001) were significantly associated with higher error scores, indicating worse cognitive performance. Female sex (B = + 0.333, *p* = 0.012) and lower functional independence as measured by the Barthel Index (B = −0.040, *p* = 0.002), also showed significant associations. The model accounted for 27.7% of the variance (adjusted *R²* = 0.277).

#### Memory Impairment Screen (MIS)

For the MIS total score, age (B = −0.015, *p* = 0.170) did not show a significant association. The total number of chronic active pharmaceutical ingredients (B = −0.044, *p* = 0.019), and cognitive reserve (B = + 0.032, *p* = 0.033) were significant predictors, while female sex, diabetes and independence in basic and instrumental activities of daily living as measured by the Barthel Index and the Lawton and Brody Scale showed nonsignificant associations. The model explained 9.9% of the variance (adjusted *R²*=0.099).

#### Semantic Verbal Fluency (SVF)

In the SVF model, higher age (B = −0.178, *p* < 0.001), female sex (B = −2.361, *p* < 0.001) and lower cognitive reserve (B = 0.405, *p* < 0.001) were associated with lower verbal fluency scores. The presence of hypertension, depression, low independence and the number of active principles showed a nonsignificant association. The overall model accounted for 27.8% of the variance (adjusted *R²* = 0.278). 

## Discussion

This cross-sectional study conducted in community pharmacies examined how different cognitive screening tools and cut-offs classify community-dwelling older adults, in a non-clinical, real-world setting. As no post-assessment clinical diagnostic confirmation was available, the results are presented as screening classifications (positive and negative) and comparative performance across instruments, not as clinical diagnoses. Therefore, our findings illustrate substantial variability in the classification of low cognitive performance or suspected cognitive impairment across different screening instruments and thresholds, rather than true prevalence or clinically confirmed cognitive impairment. This study also identifies in exploratory analyses age and low cognitive reserve as factors associated with poorer cognitive performance in this non-clinical setting, as previous evidence largely described in several previous studies [29–32]. Using the conventional Montreal Cognitive Assessment (MoCA) threshold of < 26, more than two-thirds of participants screened positive for suspected cognitive impairment. In contrast, the prevalence dropped to 27% with the more stringent cut-off of < 21, and fell below 10% when assessed with Short Portable Mental Status Questionnaire (SPMSQ), Memory Impairment Screen (MIS) or Semantic Verbal Fluency (SVF). The prevalence estimates observed in our sample fall within the broad range reported in previous studies, although direct comparisons are complicated by differences in instruments, cut-offs and settings. For example, Chun et al. reported substantial variability in the proportion of older adults classified as having mild cognitive impairment depending on the cognitive tool used and the specific scoring criteria applied [4]. Our findings that MoCA < 26 yields a much higher prevalence than lower MoCA cut-offs or other brief instruments such as SPMSQ, MIS and SVF is consistent with this literature, reinforcing the notion that methodological choices substantially influence apparent prevalence of suspected impairment in community samples [3, 9, 10, 20].

Concordance and agreement findings should be interpreted in light of the instruments’ differing scope. The MoCA is designed as a broad multi-domain screener, whereas the SPMSQ, MIS and SVF are briefer tools with a more targeted emphasis in different cognitive domains (orientation/attention and calculation; episodic memory; semantic fluency/executive retrieval). Therefore, a low Kappa under a highly sensitive MoCA threshold is not unexpected and likely reflects differences in construct coverage and classification thresholds rather than poor performance of the alternative instruments. From a public-health and service-delivery perspective, quantifying this screening discordance is relevant because these tools are used in real-world community pathways to decide who may warrant further clinical assessment.

That noted, the concordance analysis revealed that MoCA cut-off < 26 showed poor agreement with other instruments (Kappa < 0.08; <40% agreement), suggesting that this threshold may overidentify impairment relative to briefer tools. However, when applying MoCA < 21, the observed agreement with the SPMSQ, MIS, and SVF increased substantially (> 75% agreement, all Kappa < 0.14). As noted before, the poor agreement between MoCA < 26 and the other three validated tools is not unexpected considering that they emphasize partially distinct cognitive domains and use different thresholds to define abnormal performance. However, the substantial improvement in agreement when applying a cut-off of 21 suggests that a more conservative MoCA threshold may better align with briefer tools that are predominantly focused on memory, orientation and verbal fluency. This pattern supports the argument stated by previous studies, that a cut-off below 21 may be more appropriate in Spanish community-dwelling older adults, as it has been reported to improve the balance between sensitivity and specificity and may reduce misclassification [10, 20, 33–35].

The correlation matrix underscored that while instruments share some variance (moderate correlation), each assesses partially distinct cognitive domains. The MoCA, with is broader scope, captures deficits in executive functioning and visuospatial processing that the SPMSQ and MIS do not systematically evaluate. This is consistent with evidence indicating that verbal fluency and executive dysfunction often precede memory complaints in prodromal dementia [36–39]. Notably, semantic fluency correlated positively with MoCA total scores, reinforcing the contribution of lexical access and executive retrieval processes to global cognitive performance.

Beyond tests-results discordance, our exploratory analyses identified consistent factors associated with cognitive performance. Age emerged as a consistent negative predictor across all tests, confirming the pervasive effect of aging on multiple cognitive domains. Higher cognitive reserve also showed a protective association, in line with previous consolidated evidence that education and cognitively stimulating activities buffer against decline [27].

Importantly, the presence of metabolic conditions (using active medication as a proxy variable for the disease) was associated with lower test scores in univariate regression models. Even though after adjustment for age and cognitive reserve the effect was diluted, this association may suggest a potential vascular-metabolic contribution to global cognitive decline in this cohort, in line with longitudinal studies linking dyslipidemia, hypertension or diabetes with cognitive deterioration and increasing dementia risk [16, 40, 41]. Interestingly, the impact of metabolic conditions was most evident in the MoCA, but not consistently significant across the other instruments. This could reflect the greater sensitivity of the MoCA to detect executive function and attention deficits, commonly related to cardiovascular pathology [42]. This result reinforces the need to integrate cardiometabolic risk management into strategies for cognitive screening and prevention.

In this community-dwelling sample, our findings underscore the marked variability in cognitive test performance, highlighting challenges in achieving consistent impairment classification without a reference standard of confirmed diagnosis. Results suggest that applying a MoCA cut-off below 21 in this community-dwelling sample worths further profound research on the validity of this cut-off for screening purposes. Nevertheless, it cannot be denied that cognitive decline may begin up to 20 years before symptoms become noticeable, so applying a stricter cut-off could help identify participants at risk and monitor their progression [43].

These results emphasize factors associated with lower cognitive scores—such as age and reduced cognitive reserve—and their implications for interpreting cognitive data in epidemiological studies of community-dwelling older adults. Furthermore, they highlight the need for comprehensive cognitive profiling that considers multiple domains and adjusts for individual risk factors such as metabolic comorbidities and cognitive reserve.

### Strengths and limitations

The study benefits from a well-characterized community sample and the simultaneous administration of multiple validated tools. Nevertheless, limitations should be acknowledged: First, the cross-sectional design precludes casual inference. Second, although the sample size met a priori calculation for estimating prevalence, it may be relatively modest for multivariate modelling with several predictors, which can reduce the stability of odds ratio estimates and limit the precision of the findings. In addition, the low prevalence of impairment according to some instruments may have contributed to wide confidence intervals and less precise measures of concordance. As a result, multivariable regressions should be regarded as exploratory and in need of replication in larger cohorts. Third, comorbidities were mainly inferred from medication use, that could lead to misclassification due to this proxy approach, as some conditions may not be treated pharmacologically or medications may be prescribed for other indications. Although comorbidities were cross-checked through self-reported medical history during the interview and medical records provided by the participants, misclassifications could attenuate or distort associations between comorbidities and cognitive performance. Fourth, the study did not include a clinical diagnosis reference standard such as clinical neuropsychological assessment or specialist evaluation. Consequently, we were unable to estimate sensitivity and specificity of the different instruments or to determine which combination of tools would provide optimal diagnostic accuracy. Our findings should therefore be interpreted as describing patterns and associated factors in a community sample, rather than providing definitive evidence on diagnostic performance. Finally, because the brief instruments provide limited subdomain scoring and do not map one-to-one onto MoCA subscales, we could not perform a formal domain-by-domains concordance analysis; hence our comparisons focus on screening classifications and total-score correlations.

Future research should adopt longitudinal designs, incorporating biomarkers and neuroimaging that could help clarify the predictive value of different screening thresholds, and explore the cross-cultural sociodemographic and metabolic factors affecting the development of cognitive decline, extending these comparisons to cognitive-domain level analyses. Moreover, further work should be focused on studying the implications of metabolic conditions in the development of cognitive impairment, alongside developing comprehensive screening protocols in community pharmacies and primary care that account for key associated factors such as age, cognitive reserve and metabolic comorbidities.

## Conclusion

In summary, this research highlights the marked variability in the classification of community-dwelling older adults among widely used brief cognitive screening instruments and cut-offs. The findings support the adoption of a more conservative Montreal Cognitive Assessment cut-off (< 21) to improve concordance with other brief instruments in Spanish community-dwelling older adults. Additionally, the observed association between metabolic conditions and lower cognitive performance, although attenuated after adjustment by age and cognitive reserve, reinforces the relevance of cardiometabolic health in cognitive aging and highlights the importance of integrated preventive screening strategies in primary care and community settings, combining sensitive cognitive tools with cardiometabolic risk management. These associations should be interpreted as hypothesis-generating and require confirmation in larger samples with clinical diagnostic reference standards.

## Supplementary Information

Supplementary Material 1.

## Data Availability

Data are available upon reasonable request.

## Abbreviations

B: Regression coefficient
CI: Cognitive Impairment
MIS: Memory Impairment Screen
MoCA: Montreal Cognitive Assessment
SD: Standard Deviation
SE: Standard Error
SPMSQ: Short Portable Mental Status Questionnaire
SVF: Semantic Verbal Fluency
