# Supplementary material for: Identifying individuals at risk of cognitive decline: cross-sectional analysis of variability in neuropsychological test scores among community-dwelling older adults
Source: BMC Public Health. 2026 Apr 9;26:1622. doi: 10.1186/s12889-026-27246-y (PMC13191990; doi:10.1186/s12889-026-27246-y)
Supplement: Supplementary file 1 — Supplementary Material 1. [file 12889_2026_27246_MOESM1_ESM.docx]

**Appendix 1. Pearson’s correlation analysis between test scores**

|  | **MoCA** | **SPMSQ** | **MIS** | **SVF** |
| --- | --- | --- | --- | --- |
| **MoCA** | 1 | -.6048^***^ | .3510^***^ | .4741^***^ |
| **SPMSQ** | -.6048^***^ | 1 | -.3645^***^ | -.3849^***^ |
| **MIS** | .3510^***^ | -.3645^***^ | 1 | .2474^***^ |
| **SVF** | .4741^***^ | -.3849^***^ | .2474^***^ | 1 |

****: correlation is significant at p<0.001 level (two-tailed)*
